# Bisphenol, npcRNAs and Utero-Ovarian Feed-Back Control of Breast Cancer Chemosensitivity

**DOI:** 10.1289/ehp.0900983

**Published:** 2009-08

**Authors:** Aysegul Yarpuzlu

**Affiliations:** Department of Public Health and Biotechnology, Ankara University, Ankara, Turkey, E-mail: aysegul.yarpuzlu@medicine.ankara.edu.tr

In the February 2009 issue of *Environmental Health Perspectives*, La Pensee et al. (2009) postulated that bisphenol A (BPA), at nanomolecular doses, confers chemo resistance in estrogen receptor (ER)-α–positive and –negative breast cancer cells. Certainly, drug resistance is well-known to be an important complication in a variety of cancer chemotherapy options. Several molecular mechanisms have been suggested to explain the onset of drug resistance. Determining the exact mechanism in a particular case is challenging both at the clinical and preclinical research levels because the genome-wide and proteomic approaches to mechanistic studies are still at a developing stage (Zhang and Liu 2007).

With regard to cisplatin, doxorubicin, and vinblastine cytotoxicity, there are proposed mechanisms of cytotoxicity in breast cancer cells and cell line s other than ER or receptor-mediated molecular signals. These mechanisms involve not only apoptosis pathways but also other regulatory, functional, and structural mechanisms of phenotypic expression in breast cancer models that could interfere with androgen receptor-mediated transcriptional activities (Aube et al. 2008). In addition, after the invention of microarray systems, more focus has been placed on the large number of human transcripts that have been described but do not code for proteins, such as nonprotein coding RNAs (Mallardo et al. 2008). These may include subfractions of small (microRNAs, small nucleolar RNAs) and long RNAs (anti-sense RNA, double-stranded RNA, and long RNA species) that function as regulators of other mRNAs at the transcriptional and post-transcriptional levels and control protein ubiquitination and degradation, with possible other roles yet to be elucidated. Various species of nonprotein-coding RNAs (npcRNAs) have been found to be differentially expressed in diverse types of cancer, including breast cancer subtypes (Mallardo et al. 2008). Because BPA is a highly reactive chemical, it would not be surprising if it interacts with some of these npc RNAs that could mediate ER response.

Recent reports from other laboratories have tended to support a role of npcRNAs in BPA-mediated mechanisms involved in breast cancer and a possible physiochemical interaction of BPA with estrogen and non-estrogen-mediated chemosensitivity-inducing pathway elements. For example, Hong et al. (2006) used expression micro array technology to predict hormone-responsive activities in response to estrogen and endocrine disruptors. According to these authors, the expression levels of only 555 genes (7.42%) among the 7,636 genes spotted on microarray chips were enhanced by > 2-fold after treatment with estradiol (E_2_), suggesting that direct or rapid response to E_2_ is widespread at the mRNA levels in these genes. Hong et al. (2006) observed that elevated expression levels of the genes (over 2-fold) were induced by BPA (8.26%) in the uterus of immature rats. Examples of differentially expressed representative genes include calbindin-D9k (vitamin D-dependent calcium-binding protein), oxytocin, adipocyte complement related protein (30 kDa), lactate dehydrogenase A, and calcium-binding protein A6 (calcyclin). The mRNA levels of these genes were also increased in various phases of the menstrual cycle. This study in rats (Hong et al. 2006) supports the possibility of distinct effects of endogenous E_2_ and environmental endocrine-disrupting chemicals in the uterus of women. Involvement of these gene transcripts, which are present in breast, uterine, and ovarian tissues, in the environment–endocrine inter action suggests the possibility of a utero-ovarian feedback control of breast cancer chemo sensitivity effected by npcRNAs.

Sladek and Somponpun (2008) studied the effect of vasopressin (VP) on the reproductive cycles of humans; their results suggest the involvement of multiple types of ERs in the VP-mediated G-protein coupled response (Hong et al. 2006). VP, acting through fluid-electrolyte mechanisms, may have a role in the mechanism of breast cancer initiation and progression, which may be prone to regulatory impacts through npcRNAs involved in the replication of dysregulating pathways of the mammary epithelium.

These observations suggest that there may be a utero-ovarian feedback control mediated by ERs on the uterus that cross-talk with vasoneural pathways; the feedback control may mediate estrogen involved in chemoresponsive pathways of breast cancer. Furthermore, these pathways may be regulated by noncoding npcRNAs whose functions may be physiochemically modified by environmental toxicants such as BPA and other related chemials.

Since the forum titled “Bisphenol A: An Expert Panel Examination of the Relevance of Ecological, *in Vitro* and Laboratory Animal Studies for Assessing Risks to Human Health” in Chapel Hill, North Carolina, on 28–30 November 2006 (Keri et al. 2007), there has been no meeting convened to discuss environmental endocrine disruptors, particularly as it relates to BPA and related chemicals. I hope that a future review panel will assess the literature on both animals and humans and evaluate the role of BPA in carcinogenesis. Such an assessment should also include recommendations for future areas of research.

## Editor’s note

In accordance with journal policy, LaPensee et al. were asked whether they wanted to respond to this letter, but they chose not to do so.
